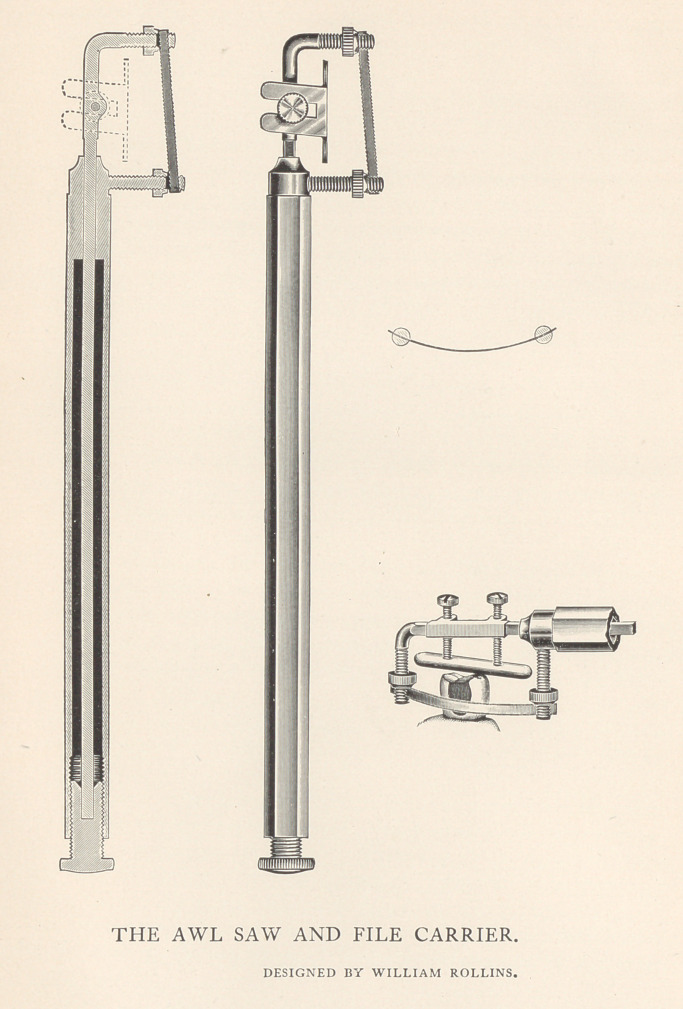# Dental Surgeons in the Army and Navy

**Published:** 1899-12

**Authors:** George T. Baker

**Affiliations:** Boston, Mass.


### International Dental Journal.

Vol. XX. December, 1899. No. 12.

### Original Communications.[1]

### DENTAL SURGEONS IN THE ARMY AND NAVY.[2]

BY GEORGE T. BAKER, D.D.S., BOSTON, MASS.

It is not the purpose of this paper to discuss the best method of organizing a dental corps in the army and navy, but simply to call attention to the importance of the subject, and, if possible, enlist the hearty co-operation of the members of this Society. During the past year much thought has been given to the matter, and the unusual interest taken by every one in military and naval affairs has perhaps given impetus to the movement.

Without doubt the time is coming when the men in both army and navy will enjoy the services of skilful dentists; it is also true that there is almost no opposition to the end in view, the only delay being that caused by hesitation as to just what should be done and just how best to do it.

In order that we may appreciate the need of such service, let us for a moment glance at the organization of our army and navy, and especially the medical departments in each, for to these departments, if any, must a dental corps look for encouragement and support.

---

[1] The editor and publishers are not responsible for the views of authors of papers published in this department, nor for any claim to novelty, or otherwise, that may be made by them. No papers will be received for this department that have appeared in any other journal published in the country.

[2] Read before the Massachusetts Dental Society, June, 1899.

Our army in time of peace is divided into eight departments,—viz., Department of the East, of Missouri, of Dakota, of the Platte, of Texas, of Colorado, of California, and of Columbia. These departments are subdivided into posts or stations, about a hundred in all, garrisoned by a varying number of men. Here are stationed the men under their officers, the latter in most cases accompanied by their families and civilian *attachés* of the army. The civilian *attachés* comprise the families of the officers and enlisted men, servants, employees of the various departments and their families, and all persons not in the *personnel* of the army who are allowed to reside at military stations or to accompany military commands. The number of the non-combatants during the year 1897 was as follows (*vide* "Report of Surgeon-General of the Army"): Adult males, 2178; adult females, 5710; children, 5968; total, 13,856. This number added to the total number of the army, 25,417, makes a grand total of 39,273, including over ten thousand women and children. When our army is increased to sixty-five thousand, the grand total must be in the vicinity of one hundred thousand persons living in army posts and stations, often many miles distant from civilized communities.

In the navy the conditions are much the same, only the officers, except on home stations, are not accompanied by their families. Here the various *stations* correspond to the *departments* of the army, as the North Atlantic Station, the Pacific, the South Atlantic, the European, the Asiatic, and one other, the Northwestern Lakes. Then comes what correspond to the posts of the army. These include the navy-yards, marine barracks, etc., and the different ships, each attached to this, that, or the other station.

The men in the army are enlisted for five years, and in the navy for four, and inducements for re-enlistment are held out, such as increased pay, rank, etc., so that many of the men, like the officers, serve a long period of years, or for life.

While the line officers have always been educated and trained in the Military Academy at West Point, or the Naval Academy at Annapolis, the medical officers have always been appointed from civil life, after a rigid and exacting examination, both physically and professionally. The applicants are plenty, so that it may be said of them that "while many are called, but few are chosen."

In both departments are men of scholarly attainments and world-wide reputation. To show with what care the appointments for the army are made, it may be interesting to know that during the year ending June 30, 1898, of one hundred and eighty-one applicants authorized to appear before the Examining Board, only one hundred and thirty-one appeared, and but nineteen passed; and during the same period for the Medical Department of the navy, out of two hundred and forty-eight applicants, only sixty-five appeared, and but seventeen passed. This makes the successful candidates for the army about fifteen per cent., and for the navy about twenty-six per cent. of those examined. After receiving his commission the army surgeon enters the Army Medical School at Washington (established in 1893), and for six months a special course is taken in the following studies: Duties of Medical Officers, Military Surgery, Military Medicine, Military Hygiene, Sanitary Chemistry, Clinical and Sanitary Microscopy, Hospital Corps Drill and First Aid. In addition, instruction in riding is given by a cavalry officer.

Much might be said of the hospitals provided with every facility for the best possible treatment of the sick and injured. Recently ambulance ships have been added to the navy, the "Solace" being the first of her class.

In 1887 the hospital corps of the army was organized. This corps consisted, January 31, 1898, of over seven hundred men, who perform service as ward-masters, cooks, nurses, attendants in hospitals, as stretcher-bearers, litter-bearers, and ambulance attendants in the field, and such other duties as may by proper authority be required of them.

There is also a hospital corps of the navy recently organized by Act of Congress and approved by the President June 17, 1898, and nearly all the hospitals are now supplied with trained nurses, and in many are apprentices undergoing instruction.

While all these precautions for the health and safety of the men seem excellent, there appears to be something lacking. Apparently it is taken for granted that the men have perfect teeth and are immune to all dental troubles, but, as is generally known, the rank and file of our army and the seamen of our navy are from a class who, while strong and rugged, often suffer acutely from defective teeth. To many of them modern dentistry, with its relief from pain and suffering, is an unknown quantity, and the only remedy known is extraction. This primitive method of relief supplied by the medical departments in both army and navy is the same to-day as it was one hundred years ago, and must result in the long run in great loss of time and the ruthless extraction of comparatively sound teeth. Such wholesale extraction is malpractice, and while it may have been necessary in early times it is to-day without excuse.

It is recorded that Erasistratus once deposited in the Temple of Apollo at Delphos a leaden forceps, to prove that only those teeth ought to be removed which are loose or relaxed and for which a leaden forceps will suffice. Possibly if some modern Erasistratus would deposit a similar forceps in the Army Medical Museum at Washington the lesson would not go unheeded.

The theory that a tooth should be extracted because it aches has given way to the more advanced idea that it can and should be saved; or, better still, on the principle that "an ounce of prevention is worth a pound of cure," the tooth should not be allowed to ache, but by frequent examinations dental caries when present should be early discovered, the cavity excavated and filled, thus avoiding the more difficult process of treatment in order to save the tooth. Such work can be done only by men educated and trained in the science and art of dentistry, and such education and training requires much time and patient study. Not only the mind must comprehend what is to be done, but the hand and fingers must think as well.

The organization of a dental corps as an auxiliary to the Medical Departments in both army and navy would tend to increase the efficiency and promote the general health of the men, and in the case of the army not only the men, but the officers and their families, would derive great comfort and benefit from such an organization. The need seems to be almost imperative, for the uninterrupted tour of duties makes it well-nigh impossible for any dental treatment unless supplied close at hand by the Medical Department.

The need of such a dental corps has been recognized for a long time. At the Columbian Dental Congress a committee was appointed to report on the matter. There were representatives from the United States, from the principal European countries, and from four of the South American republics, and though the report was not especially encouraging, it was recommended that the matter be brought to the attention of the Surgeon-General of the army every year.

As is generally known, the matter was before the last Congress, and though the bill introduced by Mr. Otey, of Virginia, was not favorably received, there is reason to believe that the Hull Bill

THE AWL SAW AND FILE CARRIER.

DESIGNED BY WILLIAM ROLLINS.

will meet with better success. In the mean time let all strive to urge upon those in a position to help the movement the importance of the matter from a humanitarian point of view. Dental surgeons should be represented in our army and navy and in our State militias, as well as upon the staffs of our various city and State hospitals. If our various State societies will heartily support this movement, and encourage the committee appointed by the National Dental Association at the recent meeting at Omaha, who now have the matter in hand, it will only be a question of time when the whole matter will be brought to a successful issue.

## Figures and Tables

**Figure f1:**